# A Test of the Self-Medication Hypothesis Using a Latent Measurement Model: Are Stress and Impaired Control over Alcohol Mediating Mechanisms of Parenting Styles on Heavy Episodic Drinking and Alcohol-Related Problems among University Students?

**DOI:** 10.3390/bs14050384

**Published:** 2024-05-02

**Authors:** Felix B. Muniz, Elena Kalina, Julie A. Patock-Peckham, Sophia Berberian, Brittney Fulop, Jason Williams, Robert F. Leeman

**Affiliations:** 1 Department of Psychology, Arizona State University, Tempe, AZ 85287-1104, USA; julie.patock@asu.edu (J.A.P.-P.); slberber@asu.edu (S.B.);; 2 Department of Health Education and Behavior, University of Florida, Gainesville, FL 32611, USA; ekalina@ufl.edu; 3 RTI International, Research Triangle Park, NC 27709, USA; jawilliams@rti.org; 4 Bouvé College of Health Sciences, Northeastern University, Boston, MA 02115, USA; r.leeman@northeastern.edu

**Keywords:** latent measurement model, stress, impaired control over drinking, heavy-episodic-drinking, parenting styles, alcohol-related problems

## Abstract

Introduction: The self-medication hypothesis (SMH) suggests that individuals consume alcohol to alleviate stressful emotions. Still, the underlying mechanisms between stress and heavy episodic drinking remain to be explored. Impaired control over drinking (IC) reflects a failure of self-regulation specific to the drinking context, with individuals exceeding self-prescribed limits. Parenting styles experienced during childhood have a lasting influence on the stress response, which may contribute to IC. Method: We examined the indirect influences of parenting styles (e.g., permissive, authoritarian, and authoritative) on heavy episodic drinking and alcohol-related problems through the mediating mechanisms of stress and IC. We fit a latent measurement model with 938 (473 men; 465 women) university students, utilizing bootstrap confidence intervals, in Mplus 8.0. Results: Higher levels of authoritative parenting (mother and father) were indirectly linked to fewer alcohol-related problems and less heavy episodic drinking through less stress and IC. Maternal permissiveness was indirectly linked to more alcohol-related problems and heavy episodic drinking through more stress and, in turn, more IC. Impaired control appeared to be a mediator for stress and alcohol-related problems. Conclusions: Maternal permissiveness contributes to the use of alcohol to alleviate stress. Thus, reducing stress may reduce problematic heavy drinking and alcohol problems among emerging adults with high IC who may also have experienced permissive parenting. Stress may exacerbate behavioral dysregulation of drinking within self-prescribed limits.

## 1. Introduction

When life is unpredictable, uncontrollable, or overwhelming [1,2], people often report feeling stressed, especially when family relationships are less than ideal [3]. Stress is the feeling that there are insufficient resources to cope with life events [2,4]. Family-related stress is often inescapable, as the relationships are long-term. As feelings of stress can be overwhelming, some individuals lose their sense of self-control [5]. In some situations, heightened stress can reduce self-control around alcohol [6,7,8,9,10]. Two key outcomes of reduced self-control around alcohol (i.e., impaired control over alcohol) are heavy episodic drinking and alcohol-related problems [11,12], which are particularly common among college students [13]. One study reported that about 29% of college students had engaged in heavy episodic drinking in the past month [14], which contributes to about 15% of college students experiencing alcohol use disorder (AUD) [15]. A growing area of the literature suggests that parenting styles have a lasting influence on the stress response [16] and may contribute to impaired control over alcohol e.g., [17,18,19]. Further, parenting and parenting styles are potential points of intervention to prevent eventual heavy episodic drinking and alcohol-related problems among offspring who become college students [20]. The current study is an exploration of the indirect effects of parenting styles on heavy episodic drinking and alcohol-related problems among college students through mechanisms of stress and reduced self-control around alcohol (i.e., impaired control over alcohol).

### 1.1. Attachment, Parenting Style, and Stress Response from a Developmental Perspective

The emotional socialization theory suggests that early parent–child attachment, or lack thereof, is a key dimension of the environment that “sets the stage” for overall child emotional adjustment [16]. The quality of the parent–child relationship interacts with the child’s stress response and temperament from as young as the age of three [21]. Boldt et al. [22] assessed attachment at 2 years of age and found that those that were more securely attached were better able to regulate negative emotions at ages 10–12. Importantly, positive attachment prior to age three is critical for the development of a healthy sympathetic nervous system (SNS) and hypothalamic pituitary adrenal (HPA) response [23]. Romanian orphans in institutional care (18 months) without clear attachment to a caregiver were more likely to experience a blunted stress response (i.e., cortisol reactions) to the Trier social stress test (TSST), a social evaluative acute stressor test, [24] at age 12, while orphans with supportive/reliable foster care experienced a normal stress response [23]. Positive attachments continue to buffer stress beyond infancy and throughout middle childhood as well. When 7–12-year-old girls were administered the TSST-C (child version) [25], those who were able to speak to their mother returned to baseline cortisol faster than those whose mothers were unavailable after the TSST-C was administered [26]. These studies point to how parent–child relationships influence the development of stress regulation throughout childhood, as well as through early adolescence. Parenting styles, while more distal, contribute to the quality of parental bonds and attachment, even in late adolescence and emerging adulthood [17,18,27]. Conceivably, parenting styles may have a lasting influence on offspring stress and other related factors throughout development.

A well-validated categorization of parenting styles includes authoritarian, authoritative, and permissive parenting [28]. Authoritarian styles are typically described as controlling; the parent provides unyielding direction without warmth or flexibility, which may leave the child feeling insecure and inferior [29]. Moreover, authoritarian parenting has been linked to increases in internalizing behavior [30,31]. Authoritarian fathering is directly linked to both depression among offspring [17,18] and neuroticism among sons [25]; sons also show increased stress later in life [32]. Having an authoritarian mother has been associated indirectly with dysregulated drinking through increased self-concealment [33], as well as indirectly associated with increased depression, and self-medication motives for drinking through the mediating mechanism of higher levels of perfectionism discrepancy [34].

Social learning theory [28,35] suggests that parents exhibiting authoritative parenting styles (high warmth with balanced limit setting) model healthier ways of handling emotions and other daily challenges [28,36,37]. This style is later emulated by preteens [38] as well as emerging adults [39]; this is especially evident regarding alcohol use [40]. Authoritative parenting is linked to several protective factors (for a review see [41]) including better self-regulation [39], greater levels of achievement, high standards [34], as well as fewer depressive symptoms, reduced impulsivity [42], and less monthly drinking [43,44]. The permissive parenting style consists of a lack of structure or guidance that allows progenies to make decisions usually reserved for adults [45]; it is linked to lower self-regulation [39] and academic achievement, as well as greater impulsivity [42,45], delinquency, and alcohol use [46]. The consensus is that authoritative and permissive parenting are associated with warmer bonds/attachment to parents, while authoritarian parenting is more associated with the occurrence of internalizing symptoms [31]. Based on the research evidence presented above, parenting styles differ in their influence on child alcohol risk based on the gender of the parent. Thus, the current study sought to understand the indirect influence of parenting styles by parent gender on heavy episodic drinking and alcohol-related problems through mechanisms of stress and impaired control over alcohol (IC; dysregulated drinking).

### 1.2. Congruence of Parent and Child Reports of Quality/Style of Parenting

Informant discrepancy between young children and parent reports of parental rearing practices has been well-documented, and varies widely by interviewing technique and/or whether child welfare/social services are involved (see [47] for a review). Parents under investigation for abuse tend to provide more positive ratings than their children [48]. Yet, warm parenting styles [authoritative (warm with balanced limits) and permissive (warm yet unstructured)] were related to increased health behaviors such as bedtime toothbrushing and consumption of milk among Korean children aged three to six [49]. Despite some discrepancies between very young children and parents when social services are involved, there is more convergence of mother, father, and child perceptions of parenting in adolescence [50].

As children get older, parents will notice that alienation increases, and trust and communication decrease, starting as early as sixth grade through high school [51]. Less trust with both parents in sixth grade and increased alienation from mothers in high school have been associated with more depression by grade 12 [51]. Nevertheless, Keizer et al. [52] found that the perceived quality of mother–adolescent attachment relationships was positively associated with offspring self-esteem across three years (ages 13, 14, and 15) for both daughters and sons, with father–adolescent attachment relationships affecting only their daughters and not their sons. While teenagers (ages 13–15) may increase their time spent with friends and romantic partners, their perceptions of the quality of their relationships with their parents are highly important for how adolescents think about and judge themselves [52]. The internalization of positive morals and self-esteem was found to be aided by a warm authoritative parenting style among adolescents from Spain, Portugal, and Brazil [53] (N = 2091). Harsh control, found in the authoritarian parenting style, was associated with increased anxiety, depression, and suicidal ideation among adolescents, while authoritative parenting (warmth with balanced control) was found to be a buffer against internalizing symptoms (see [54] for a review). In fact, Parra et al. [55] demonstrated that the influence of parenting style on children’s well-being is indeed sustained during emerging adulthood. This is consistent with findings from the National Survey of Midlife Development in the United States (MIDUS, 1995, N = 4244; 25–74 years of age; mean age of 54 years), which revealed that adults who remembered authoritative rather than authoritarian parenting reported greater psychological well-being and life satisfaction [38], with uninvolved parenting being more associated with substance use.

### 1.3. The Self-Medication of Distress by Using Alcohol to Cope

The self-medication hypothesis (SMH) theorizes that individuals consume alcohol as a means of medicating their mental distress. A substantial amount of the literature regarding the SMH has explored how increased coping motives surrounding alcohol are associated with more frequent alcohol-related problems [56,57,58,59,60]. While social norms are the best predictor of alcohol consumption, coping motives are a better predictor of alcohol problems among heavy-drinking college students [61] (N = 818). For instance, Windle and Windle [60] (N = 1205) found that stressful events and coping motives prospectively predicted greater alcohol involvement—especially problematic use over time (5 years)—among middle-aged adults. Internalizing symptoms such as anxiety and depression were linked to increased coping motives for drinking, and were particularly likely to alleviate low moods among adolescents of a lower socio-economic status [58] (N = 3957). While coping motives are more common among women [62], they have been found to be strongly associated with increased alcohol problems among men with elevated depressive symptoms [57].

Parenting appears to influence the motive to cope with alcohol across several different cultures. Insecure attachment to one’s parents and early life stress was directly associated with increased motivation to use alcohol among 256 adult men from Korea [63]. Further, Voce and Anderson [59] found interactions between parental permissiveness toward alcohol and motives for alcohol (conformity and coping) among American high school students. Even among students with low coping motives for drinking, higher parental permissiveness was associated with increased quantity and frequency of drinking alcohol. Moreover, Bitsoih et al. [64] (N = 612) found that both emotional and sexual abuse at the hands of caregivers/parents were directly associated with increased coping motives and, in turn, indirectly associated with increased impaired control over drinking (IC; drinking longer or more than intended) among college students.

A plethora of high-quality research has already been conducted exploring coping motives as a potential mediator of parenting influences on alcohol use and related problems. Yet, little research has been conducted on IC as a potential mediating mechanism of the indirect influences of parenting on alcohol use and related problems via stress. IC describes the tendency to surpass self-prescribed limits on alcohol consumption [65] and is associated with alcohol-related problems and heavy episodic drinking among young adults. This includes prospective evidence [12,66]. The SMH posits a link between stress and IC [67,68,69]; yet, this link has never been explored in the literature with parenting styles as an indirect contributor. We feel this is important to study because Canning et al. [70] found a direct link between coping motives and impaired control over drinking, and IC reflects self-control regarding drinking behaviors [39,71]. While recent experimental work [6] and survey work [72,73] has found a direct link between stress and IC, no prior research has yet considered the role of parenting influences on this association.

### 1.4. Stress and Impaired Control over Drinking

IC is often considered to be one of the first indicators of an alcohol use disorder starting to develop [13,65,71]. It is also recognized as a failure of self-regulation specific to the drinking context [39,42,45]. Cross-sectional research has shown that IC is directly linked to heavy episodic drinking and alcohol-related problems [64,70,74], and it has also been shown to prospectively predict risk for alcohol use disorders (AUDs) [12,66]. Despite the role of IC in problematic alcohol use, little research has examined the role of stress on this association.

Leeman and colleagues [75] were the first to examine an association between IC and stress, in which previous 6-month IC was correlated with stressful life events. This finding suggested that IC may increase with increased stress during one’s life. Since then, more recent cross-sectional work has found that stress is directly linked to more IC [72,73]. IC appears to be related to stress in different contexts. For example, Berberian et al. [72] examined IC and stress in the context of loneliness experienced during childhood, while Kalina et al. [73] examined IC and stress in the context of relationship-contingent self-esteem (i.e., one’s self-worth being vulnerable to the status of one’s romantic relationships) [76]. Further, recent experimental work showed that social evaluative stress (TSST) was related to an increased likelihood of violating behavioral IC instructions during an alcohol administration session [6]. These results collectively corroborate the SMH, suggesting that heightened stress may lead to a lack of self-control around alcohol. Suboptimal parenting constitutes another set of environmental experiences that may influence both stress and self-control around alcohol. In fact, a recent study identified self-control as a mediator in the relationship between parenting styles and problematic behavior [77]. Yet, no prior research has considered relationships among parenting styles, stress, and IC.

### 1.5. Latent Variable Modeling for Measurement

Our study also aspires to contribute to methodological literature. Namely, this study serves as the first exploration of a latent variable model of stress and IC as mediating mechanisms in the relationship between parenting styles and alcohol outcomes. In the field of psychology, we commonly score measurement scales by either summing all items or dividing the sum by the total items to obtain an average score. Sum and average scores are susceptible to error in calculation and often the decision to select either scoring option is arbitrary, of which both issues contribute to the ongoing replication crisis. When using sum or average scores, the underlying statistical assumption is that each item is equally related to the overall construct of interest [78]. Since each item in a sum or average score is weighted equally in the construct, the factor loadings for each item are assumed to be equal, resulting in model restriction. In contrast, a latent measurement model allows the factor loading of each item to freely estimate within the model and thus more accurately represents each participant’s score on the construct. This method allows for validation of the unidimensional factor structure of a construct, which sum and average scores cannot validate [78]. Further, few studies report validity or reliability statistics [78], which diminishes the credibility of the traditional methods used. In contrast, a latent measurement model enhances the reliability and validity of the measurement methods. Given the benefits above, the current study utilizes a latent measurement model for both the stress and IC scales to confirm their unidimensional factor structures and to increase the reliability and validity of the measures which are of essential interest to our study.

### 1.6. Hypotheses

**Figure 1:**
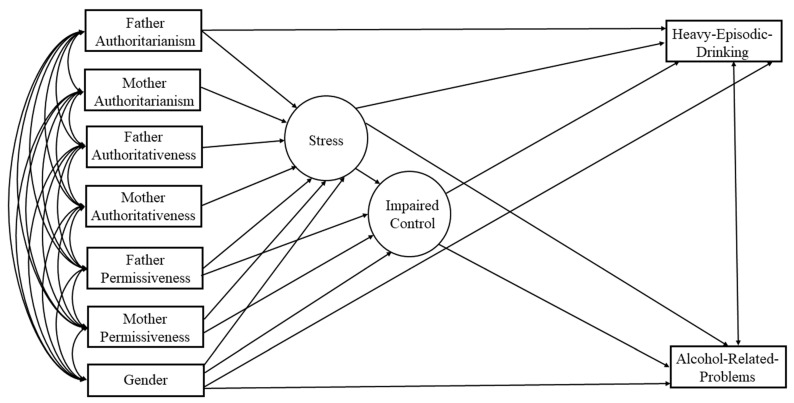
Conceptual model. In our conceptual model we have included every path that we have tested among the different variables in the model.

Our conceptual structural equation model (SEM; Figure 1) comprises several hypotheses. Based on the SMH, we expected authoritarian parenting to be positively related to stress. Likewise, we expected the authoritarian parenting style to be indirectly related to increased heavy episodic drinking and alcohol-related problems. Because authoritarian parenting is known to increase internalizing behavior [30], we predicted stress to mediate the relationship between authoritarian parenting and IC. Next, due to a lack of structure for children, we predicted permissive parenting would be positively related to stress. In addition, we anticipated that permissive parenting would be indirectly related to increased heavy episodic drinking and alcohol-related problems through more stress and more IC. In contrast, authoritative parenting was expected to be negatively linked to stress, since this parenting style allows structure for children, with room to grow and without overwhelming offspring with expectations of strict obedience. Finally, we predicted that authoritative parenting would be indirectly related to less heavy episodic drinking and fewer alcohol-related problems through less stress and less IC.

## 2. Methods

### 2.1. Participants

The sample consisted of 938 participants (473 men; 465 women) with a mean age of 19.88 (SD = 2.79). Our sample identified as 54% White/Caucasian, 22% Asian, 15% Hispanic/Latinx, 5% African American, 1% Native American, and 3% other. Participants were recruited from a large university for credit in their psychology classes, with all procedures approved through the local institutional IRB.

### 2.2. Measures

1. The Parental Authority Questionnaire (PAQ) [36,37] included a 60-item measure (30 per parent) with 10 questions each based on Baumrind’s [28] prototypes of parenting styles, and separated by mother and father. Items were summed to create a variable for each parenting style and each parent (i.e., mother and father). Responses were on a 5-point Likert scale (1 = strongly disagree to 5 = strongly agree). A sample authoritative item was “As I was growing up my mother/father directed the activities and decisions of the children in the family through reason and discipline”. A sample authoritarian item was “As I was growing up my mother/father often told me exactly what she/he wanted me to do and how she/he expected me to do it”. Lastly, a sample permissive item was “While I was growing up my mother/father felt that in a well-run home the children should have their way in the family as often as the parents do”. The α reliabilities in this sample were as follows: mother permissive = .77, father permissive = .78, mother authoritarian = .84, father authoritarian = .88, mother authoritative = .83, and father authoritative = .87.
2. The Perceived Stress Scale (PSS-10) [1,2] included 10 items on a 5-point Likert scale (0 = never to 4 = very often). Example items included “In the past month, how often have you been upset because of something that happened unexpectedly?” and “In the last month, how often have you felt difficulties were piling up so high that you could not overcome them?”. The α reliability for this sample was .79.
3. The Impaired Control over Alcohol Use Part III (ICS) scale [65] included 10 items that assessed current beliefs about one’s own drinking in a 5-point Likert scale format (1 = strongly disagree to 5 = strongly agree). Sample items included “I would have difficulty limiting the amount I drink,” and “I would start to drink, even if I’d decided not to”. The α reliability for this sample was .82.
4. The heavy episodic drinking [79] survey measured occasions of heavy episodic drinking using one item. Participants responded to the question “How many times in the past year (or when you were drinking) did you drink 5 or more bottles (4 for women) or cans of beer, glasses of wine, or drinks of distilled spirits on a single occasion?” on an 8-point Likert scale (0 = never to 7 = daily or nearly daily).
5. The Young Adult Alcohol Problems Screening Test (YAAPST) [80] was also administered. We utilized the mean score from the 27-item YAAPST. Response options ranged from 0 (no, never) to 9 (40 or more times in the past year). Sample items included “Have you driven a car when you knew you had too much to drink to drive safely?” and “Have you ever gotten into trouble at work or school because of drinking?”. The α reliability was .91 for this sample.

### 2.3. Statistical Approach

Structural equation modeling with bootstrapping and full information maximum likelihood (FIML) estimation to account for missing data was fitted with Mplus version 8.0. To evaluate our conceptual model fit, the standardized root mean square residual (SRMR), root mean square error of approximation (RMSEA) [81,82], and comparative fit index (CFI) [83] were analyzed. Both direct and indirect effects were examined, with tests of indirect effects using the bootstrap technique (20,000 draws) to address non-normality in the product of coefficients [84,85,86,87]. Next, 95–99% asymmetric confidence intervals around the estimates were examined, with confidence intervals that did not include zero indicating significant indirect effects [88,89,90]. Next, latent variables were created for the stress and IC measures to increase measurement quality. We used the factor scores to show exactly how each question was related to the latent construct of stress and IC [78], instead of the conventional way of using average or sum scores to conduct the analysis.

## 3. Results

### 3.1. Model Fit

**Figure 2:**
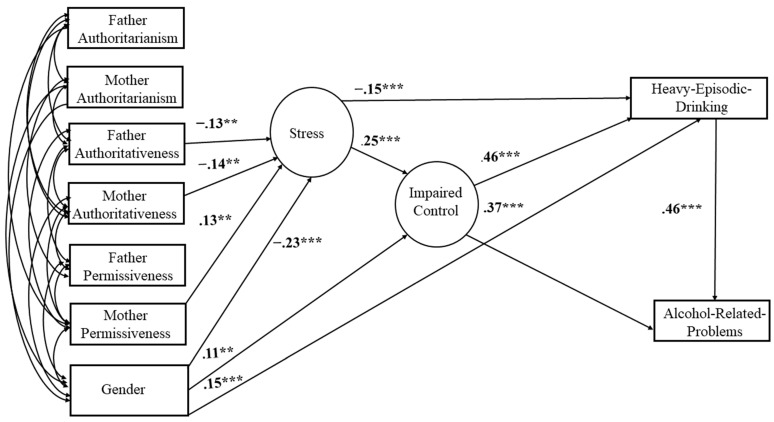
Fit model of all significant paths among the different variables in the model. ** *p* < .01, *** *p* < .001.

Although there was no way to determine acceptable cutoff values for our specific model without running a large simulation [91], high measurement quality has been shown to weaken or lower cutoff values for common model fit indices. Based on the alpha reliability coefficients for each scale, we concluded that the measurement quality is high [88]. According to the measures we described previously, model fit was acceptable by both conventional and modern standards (CFI = 0.921, RMSEA = 0.046; 90% CI (0.042, 0.049); the probability of the RMSEA < = 0.05 = 0.984; SRMR = 0.062. Please see standardized coefficients in Figure 2. Please find descriptives for all variables in the model in Table 1.

### 3.2. Measurement Model for Stress and IC

**Figure 3:**
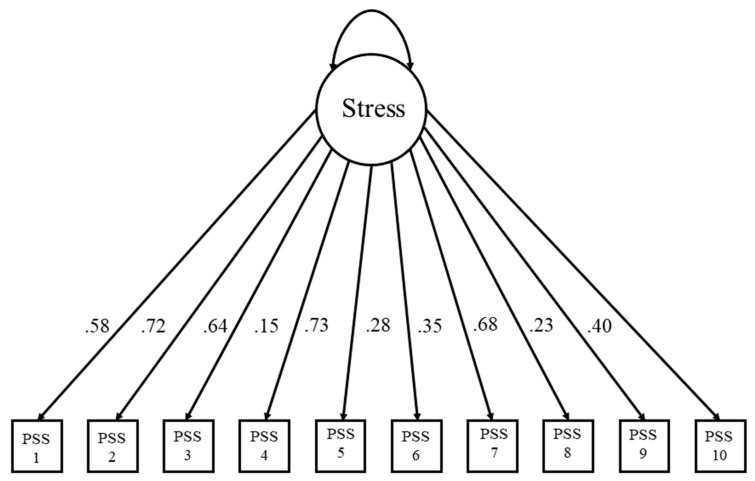
Latent variable model for stress.

**Figure 4:**
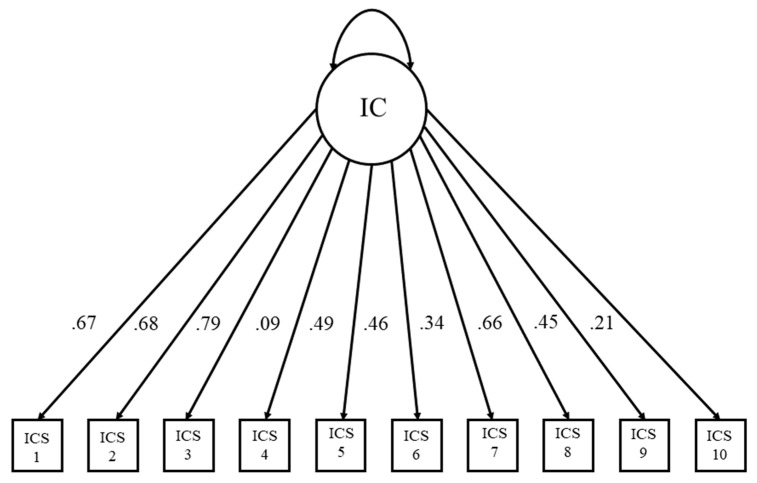
Latent variable model for impaired control over drinking (IC).

The standardized factor scores for IC ranged from 0.09 to 0.79; similarly, the standardized factor scores for stress ranged from 0.15 to 0.72. Akin to Wood [92], lower loadings were associated with reversed scored items reflecting control over drinking, rather than a lack of control concerning the IC measure. Further lower loadings were associated with a sense of calm, rather than nervous tension, in our weekly stress measure. The factor loadings for stress can be found in Figure 3, and the factor loadings for IC can be found in Figure 4. See Table 2 for factor loadings and associated items from each scale.

### 3.3. Direct Links

1. Stress: Both father and mother authoritarianism were positively related to stress, but neither was statistically significant (father β = .053, Z = 1.198, *p* > .05; mother β = .044, Z = 0.954, *p* > .05). Both father and mother authoritativeness were directly linked to lower levels of stress (father, β = −.130, Z = −3.054, *p* < .01; mother, β = −.141, Z = −3.112, *p* < .01). Father permissiveness was positively linked with stress, but was not statistically significant (β = .008, Z = .165, *p* > .05). However, higher levels of mother permissiveness were directly linked to experiencing more stress (β = .131, Z = 2.602, *p* < .01). Furthermore, men reported significantly lower levels of stress overall than women (β = −.225, Z = −6.245, *p* < .001).
2. Impaired control over drinking (IC): Higher levels of stress were directly related to higher levels of IC (β = .250, Z = 5.854, *p* < .001). Permissive parenting was positively directly related to IC but was not statistically significant (father permissiveness β = .034, Z = .816 *p* > .05; mother permissiveness β = .082, Z = 1.797, *p* > .05). Men reported higher levels of IC than women (β = .114, Z = 2.978, *p* < .01).
3. Heavy episodic drinking and alcohol-related problems: Higher levels of stress were directly related to less heavy episodic drinking (β = −.147, Z = −3.763, *p* < .001). Conversely, higher levels of stress were positively and directly related to alcohol-related problems, but were not statistically significant (β = .051, Z = 1.594, *p* > .05). Higher levels of IC were directly related to both more heavy episodic drinking (β = .456, Z = 12.593, *p* < .001) as well as more alcohol-related problems (β = .371, Z = 9.475, *p* < .001). In addition, heavy episodic drinking was directly linked to alcohol-related problems (β = .462, Z = 12.802, *p* < .001). Men were more likely than women to demonstrate heavy episodic drinking (β = .151, Z = 4.756, *p* < .001). Nevertheless, there were no differences between men and women regarding alcohol-related problems (β = .017, Z = .678, *p* > .05).

### 3.4. Indirect Effects

1. Impaired control over drinking (IC): Higher levels of parental authoritativeness were indirectly linked to less IC through less stress [father authoritativeness indirect effect = −.033, 99% C.I. (−.068, −.005); mother authoritativeness indirect effect = −.035, 99% C.I. (−.075, −.005)]. In contrast, higher levels of mother permissiveness were indirectly linked to more IC through more stress [indirect effect = .033, 95% C.I. (.008, .064)].
2. Heavy episodic drinking: Higher levels of parental authoritativeness were indirectly linked to more heavy episodic drinking through less stress [father authoritativeness indirect effect = .019, 99% C.I. (.002, .045); mother authoritativeness indirect effect = .021, 99% C.I. (.002, .049)]. In contrast, higher levels of mother permissiveness were indirectly linked to less heavy episodic drinking through more stress [indirect effect = −.019, 95% C.I. (−.041, −.004)]. Further, higher levels of parental authoritativeness were indirectly linked to less heavy episodic drinking through less stress and IC [father authoritativeness indirect effect = −.015, 99% C.I. (−.033, −.002); [mother authoritativeness indirect effect = −.016, 99% C.I. (−.036, −.002)]. Higher levels of mother permissiveness were indirectly linked to more heavy episodic drinking through more IC [indirect effect = .037, 90% C.I. (.002, .071)]. Moreover, higher levels of mother permissiveness were indirectly linked to more heavy episodic drinking through more stress and, in turn, more IC [indirect effect = .015, 95% C.I. (.000, .035)].
3. Alcohol-related problems: Higher levels of parental authoritativeness were indirectly linked to less alcohol-related problems [total father indirect effect = −.017, 99% C.I. (−.042, −.002); total mother indirect effect = −.018, 99% C.I. (−.042, −.002)]. Higher levels of parental authoritativeness were indirectly linked to more alcohol-related problems through less stress and more heavy episodic drinking [father authoritativeness indirect effect = .009, 99% C.I. (.001, .021); mother authoritativeness indirect effect = .01, 95% C.I. (.001, .024)]. In stark contrast, higher levels of authoritativeness for both fathers and mothers were indirectly linked to less alcohol-related problems through less stress and, in turn, less IC [father indirect effect = −.012, 99% C.I. (−.026, −.002); mother indirect effect = −.013, 99% C.I. (−.030, −.002)]. Lastly, higher levels of authoritativeness for both fathers and mothers were indirectly linked to less alcohol-related problems through less stress, IC, and, in turn, less heavy episodic drinking [father indirect effect = −.007, 99% C.I. (−.015, −.001); mother indirect effect = −.007, 99% C.I. (−.017, −.001)]. Hence, when IC was included in the model, authoritative parenting styles were indirectly protective of offspring experiencing alcohol-related problems.

Higher levels of mother permissiveness were indirectly linked to more alcohol-related problems through more IC [indirect effect = .03, 90% C.I. (.003, .059)]. In addition, higher levels of mother permissiveness were indirectly linked to more alcohol-related problems through more stress and more IC [indirect effect = .012, 99% C.I. (.000, .029)]. Conversely, higher levels of mother permissiveness were indirectly linked to less alcohol-related problems through more stress and less heavy episodic drinking [indirect effect = −.009, 95% C.I. (−.019, −.002)]. Yet, higher levels of mother permissiveness were indirectly linked to more alcohol-related problems through more IC and, in turn, more heavy episodic drinking [indirect effect = .017, 90% C.I. (.002, .034)]. Finally, higher levels of mother permissiveness were indirectly linked to more alcohol-related problems through more stress, increased IC, and, in turn, more heavy episodic drinking [indirect effect = .007, 95% C.I. (.000, .017)].

## 4. Discussion

The self-medication hypothesis posits a link between stress and impaired control over drinking (IC; SMH) [67,68,69], suggesting that certain individuals will consume alcohol as a means of medicating their stressful experiences. Our study shows that parenting styles can be a source of stress that influences dysregulated drinking and subsequent alcohol outcomes among young adults.

### 4.1. Stress as an Important Pathway to Impaired Control

This study adds vital knowledge to the understanding of the etiology of dysregulated drinking and how it contributes to AUDs. An abundance of the available literature indicates that IC has a direct association to maladaptive alcohol use and AUDs [11,13,39,45,64,66,70,72,73,93]. The results from the current study provide evidence that stress can alter an individual’s ability to control their impulses toward alcohol consumption. This current study is consistent with recent alcohol self-administration work with acute stress manipulations prior to ad libitum drinking [6,10]. Additionally, our model shows how the parent–child relationship may indirectly predict individual differences in IC, providing greater insight into who may be more vulnerable to AUDs through increased stress. For example, higher levels of mother permissiveness were indirectly linked to more IC through more stress. People who have highly permissive mothers may be more impulsive towards drinking than people with authoritarian mothers, because permissive mothers generally do not teach their children how to self-regulate [39]. As a result, these individuals may turn to alcohol as a stress reliever, consistent with the SMH [67,68,69]. Nevertheless, our findings regarding permissive parenting are not consistent with those of Hersh and Hussong [68], who did not have clear predictions regarding permissive parenting. Rather, Hersh and Hussong [68] focused on overbearing parents, akin to an authoritarian style, as being the most problematic for alcohol use to cope with negative feelings. This difference likely has to do with the plethora of mediating mechanisms one could test along these pathways. Parental influences on offspring outcomes are highly complex and deserve to be fully explored with numerous potential pathways to AUDs.

### 4.2. Parenting Styles

The results from the current study regarding authoritarian parenting styles are inconsistent with our SMH hypothesis [67,68,69]. Authoritarian parenting styles were unrelated to stress in our model. We predicted that authoritarian parenting would be indirectly related to more alcohol-related problems through more stress and more IC. We did not find any evidence to support this prediction. Inconsistent with what Hersh and Hussong [68] suggested, the current model revealed no relationship between the authoritarian parenting style and stress, IC, heavy episodic drinking, and/or alcohol-related problems. This could perhaps be due to the indirect influence that parenting styles have on young adult behavior, given most college students are somewhat independent from their parents. However, as mentioned in the introduction, the parent–child attachment and parenting styles have lasting effects on stress throughout development and emerging adulthood. Thus, another potential reason for this finding could be a lack of internalizing constructs in the current proposed model, such as neuroticism, depression, or anxiety sensitivity. Other research in the extant literature has found direct links between authoritarian parenting and other internalizing constructs [17,18,19]. Conceivably, stress may be a more fleeting temporary state of being and therefore may act in different and unique ways than more stable personality traits on the pathway to AUDs. Previous studies have found internalizing personality traits to mediate parental influences to drinking pathways such as depression [17], neuroticism [19], and anxiety sensitivity [94].

Our predictions and findings regarding authoritative parenting are highly consistent with the extant literature on behavioral control (i.e., self-regulation and impulsivity) [39,42,45]. Regarding both mother and father, authoritative parenting was negatively associated with stress. In turn, authoritative parenting was indirectly associated with less alcohol-related problems and heavy episodic drinking through mediation of less stress and less IC. Authoritative parents who model positive self-control also appear to have offspring who are less stressed, and thus better able to exhibit control over themselves and adhere to their own self-imposed limits for drinking behaviors [38,39].

A permissive parenting style may lead to higher levels of stress as the child grows up, due to a lack of structure as well as inappropriate boundaries. Consistent with Patock-Peckham and colleagues [42,45], permissive parenting in our model was linked with more IC. The current investigation adds to the existing literature, as it demonstrated that stress mediates the relationship between mother permissive parenting style and IC. Permissive mothers may fail to provide or demonstrate positive self-regulation skills for their children. The current study revealed a direct link between permissive mothering and stress along the IC pathway to both heavy episodic drinking as well as alcohol-related problems. This is consistent with the SMH, as individuals who struggle with feeling out of control may use alcohol to relieve that overwhelming feeling [68].

### 4.3. Latent Variable Modeling and How it Enhances Measurement of Critical Constructs

Our study also offers a novel contribution to the methodological literature because we modeled stress and IC as latent variables, which does not exist elsewhere in the existing literature. We concur with McNeish and Wolf’s [78] conclusions that simply using sum or mean scores may be insufficient for maximizing predictive abilities. By using traditional methods of sum or mean scores, researchers are assuming that each question is in fact equally related to the latent construct. The variation in factor loadings shows that this is not the case and the nuances in each construct are not captured by sum or mean scores. Marsh et al. [95] conducted a psychometric assessment of the impaired control scale (ICS-part III) with both social and treatment drinkers using confirmatory factor analyses and supported the unidimensional conclusion from the original study that developed the scale [65]. However, the factor loadings from Marsh et al. [95] did not justify using sum scores or weighing each item’s contribution to the construct equally, since the loadings ranged from 0.56 to 0.97. According to Lee’s [96] review of psychometric studies, researchers have explored one- and two-factor solutions regarding the Perceived Stress Scale—PSS-10. Based on our exploratory factor analysis, a one-factor solution best represented the internal structure of our data. To our knowledge, this study is the first to include latent variable measurement models for both the PSS-10 and ICS part III measures. This latent model technique may have enhanced our ability to observe a relationship between stress and IC. Compared to prior research using SEM to examine relationships between stress and IC [72,73], our model appears to indicate a stronger relationship between stress and IC (current model Z = 5.815; Berberian et al., [72]; Z = 4.248, Kalina et al., [73]; Z = 4.74). While the aforementioned studies [72,73] were unique, they all used similar populations of college students from the same large southwestern university as our current study. Thus, this study reflects a novel contribution to the literature, as it is the first study to use a measurement model of the ICS part III, rather than an average score, for the construct. However, replication is encouraged in an entirely new sample of participants.

### 4.4. Limitations

Although this study sought to produce insights into the development of risk factors for AUDs through mechanisms of stress and IC, the college student sample limits the current ability to generalize to the entire population. These patterns of relationships must be considered exploratory until they can be studied longitudinally. Further, while most freshmen on our campus are required to live on campus, our study is limited because we do not know whether, and/or which, students in our sample were living in dorms, apartments independently, or with their parents. Thus, our study did not allow us to determine whether the stress our participants were feeling was due to the current parenting styles of their parents, or due to the vulnerabilities of individuals to stress due to early parenting experiences. Additionally, this study used self-report methods, which can be inherently biased (e.g., social desirability, perception, etc.) and are not as verifiable as they would be in an experimental setting. Further, our study is limited because memories of parental rearing practices are not the same as a present and direct influence of parenting on offspring [47,48]. Furthermore, this study is limited because it examined both men and women in the same model. Future studies may wish to determine if these patterns of relationships are moderated by gender as the next logical step in the research process. However, it should be noted that two strengths of the current study included a nearly even split in the number of men and women in the sample, and a relatively low frequency of individuals who identified as white. Lastly, additional variables may help to further explain the relationships presented in the current manuscript (e.g., family structure; parent–child closeness) [41,97].

### 4.5. Future Directions

Further work is necessary to explore the relationship between stress and IC when it comes to negative alcohol-related outcomes. The association shown in this study (β = .25, Z = 5.854, *p* < .001) indicates the importance of examining stress and IC together, rather than only utilizing one of these constructs. Our findings suggest that stress is likely to exacerbate behavioral dysregulation of drinking within limits. Therefore, more research into this area could increase our understanding of the etiology of the role of stress regarding drinking to excess. Further, more work should also be conducted concerning contrasting parenting types and coping mechanisms for stress. It may be beneficial for future research to investigate relationships between emotional intelligence (e.g., a form of emotional self-regulation) and relationships with parenting styles and stress [98,99,100]. Likewise, it is imperative to investigate further how stress affects heavy episodic drinking, because stress was negatively directly related to heavy episodic drinking when IC was not included as a mediating mechanism in our study. This does, however, fully illustrate the importance of IC as a variable in the quest to understand the etiology of risk for AUDs.

Permissive parenting styles may be influential in the development of IC. Offspring may be more susceptible to heavy episodic drinking as well as alcohol-related problems when stressful feelings are present. Future studies should investigate the associations between permissive parenting styles and IC with other traits and environmental contexts as mediators and moderators. Further, future studies should use latent measurement modeling to enhance the validity of each question within a specific measure.

### 4.6. Conclusions

Our model identifies important targets for the prevention of AUDs in young adulthood. Families of children presenting increased stress and self-regulation difficulties may benefit from parent training interventions [101]. Parent training interventions teach parents to set appropriate and consistent rewards and consequences to change their child’s behavior. Studies show parent training interventions are efficacious for children from preschool to 18 years old and reduce symptoms of various behavior disorders, many of which are associated with alcohol use (See Long et al., [101] for a review). Additionally, young adults may benefit from AUD prevention programs focused on reducing stress and IC, such as those that encourage the use of protective behavioral strategies (PBS). PBS are cognitive–behavioral skills designed to reduce drinking and alcohol-related problems [102], and may be useful in conjunction with stress-reduction techniques. These strategies include practicing setting intentions around when and how much to drink, and recruiting others to hold you accountable to those intentions. PBS may be particularly beneficial for young adults raised by permissive parents who did not teach their children similar strategies.

## Figures and Tables

**Table 1:** Correlations, means, and standard deviations for all observed variables in the model.

M	SD	Measure	1	2	3	4	5	6	7	8	9
26.79	6.20	1. Mother Permissive	1.00								
27.21	6.61	2. Father Permissive	.47	1.00							
30.87	7.08	3. Mother Authoritarian	−.38	−.10	1.00						
32.85	7.91	4. Father Authoritarian	−.15	−.39	.33	1.00					
34.88	6.57	5. Mother Authoritative	.17	−.06	−.16	.11	1.00				
33.83	7.42	6. Father Authoritative	.01	.26	.10	−.06	.31	1.00			
0.50	.59	7. Gender	.09	.10	.05	.03	−.04	.04	1.00		
2.05	.66	8. Heavy Episodic Drinking	−.01	−.05	−.02	.04	.002	−.00	.22	1.00	
0.60	.56	9. Alcohol-Related Problems	.03	−.03	−.04	.05	.00	−.04	.13	.63	1.00

**Table 2:** Factor Loadings for Stress and Impaired Control.

Item #	Perceived Stress *(PSS-10)*	Item Stem	Impaired Control *(ICS)*	Item Stem
1	.58	Upset because of something that happened unexpectedly.	.67	Difficulty limiting amount.
2	.72	Unable to control important things in life.	.68	Drink after deciding not to.
3	.64	Felt nervous and stressed.	.79	Have more than intended, after drinking began.
**4**	**.15**	**Felt confident about ability to handle personal problems.**	**.09**	**Cut down drinking if desired.**
5	.73	Felt that things were going your way.	.49	Drinking at times when it would cause problems.
6	.28	Could not cope with all the things that you had to do.	**.46**	**Could stop drinking after 1 or 2 drinks with ease.**
**7**	**.35**	**Able to control irritations in your life.**	**.34**	**Able to stop drinking before being drunk.**
8	.68	Felt that you were on top of things.	.66	Irresistible urge to continue drinking once commenced.
**9**	**.23**	**Angered because of things that were outside of your control.**	.45	Difficulty resisting drinking.
10	.40	Felt difficulties were piling up so high that you could not overcome them.	**.21**	**Slow down drinking.**

Note. Reversed coded items are indicated with bold text. Items in the PSS-10 reflect past month experiences. Items in the ICS (part III) reflect perceived impaired control. Both the PSS-10 and IC part III item stems in the table were paraphrased from the original items from Cole (1999) [2] as well as Heather et al. (1993) [65]. Please find the full and original items in the referenced articles.

## Data Availability

Data available upon request to the corresponding author.
